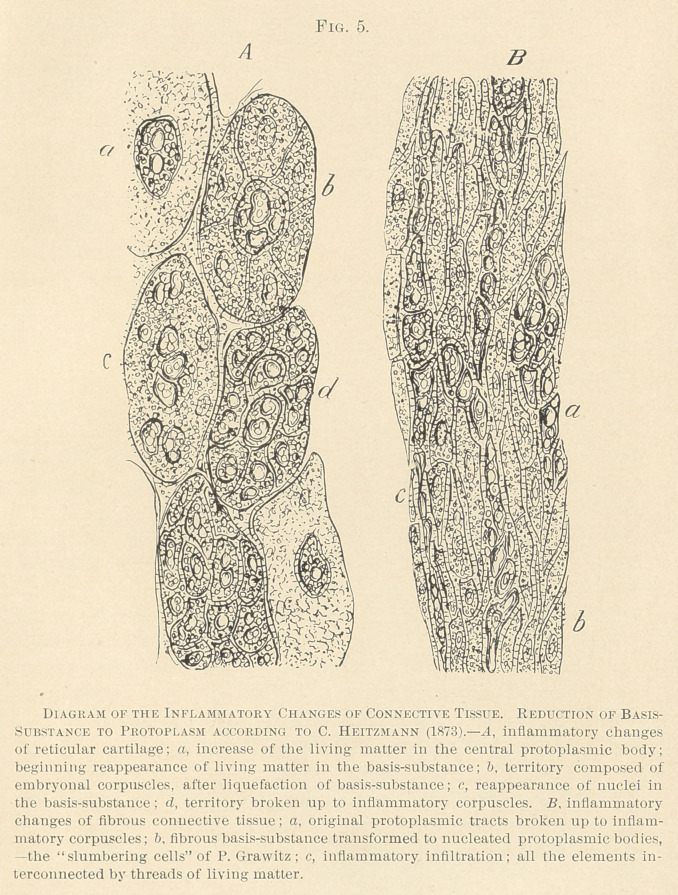# The Doctrine of Inflammation

**Published:** 1893-01

**Authors:** C. F. W. Bödecker


### International Dental Journal.

Vol. XIV. January, 1893. No. 1.

### Original Communications.[1]

### THE DOCTRINE OF INFLAMMATION.[2]

BY C. F. W. BÖDECKER, D.D.S., M.D.S.

It is with a great deal of pride that I appear before you this evening to explain and vindicate some of the latest so-called discoveries in regard to the minute changes in the tissues during the process of inflammation. The discoveries of my highly-esteemed teacher—our friend and honorary member of this society, Dr. Carl Heitzmann—are on record. It would hardly seem possible that they could have been overlooked in Germany, and yet this is the case. To some of you his ideas in regard to the minute structure of the tissues have appeared rather queer,—nay, ridiculous; but I hope the time is near at hand when our friend will receive his greatly-deserved acknowledgment for his hard labor upon the field of histological research. It was with some hesitancy that I concluded to bring before you a subject which, I am sorry to state, to some of you may seem uninteresting or impractical. If, however, we consider that we are specialists of medicine, we must admit that the more we know of the fundamental principles of histology and pathology, the better we are prepared to correctly diagnosticate and treat the various pathological disturbances presenting them-

[1] The editor and publishers are not responsible for the views of authors of papers published in this department, nor for any claim to novelty, or otherwise, that may be made by them. No papers will be received for this department that have appeared in any other journal published in the country.

[2] Read before the New York Odontological Society, November 15, 1892.

selves in our daily practice. In order that you may be able to comprehend the differences in the opinions of the authors, I shall be obliged to dwell at some length upon the several theories in regard to the minute structure of the tissues involved in the process of inflammation.

For centuries the clinical features of inflammation have been known to the surgeon. Swelling, redness, heat, pain, and impaired function were considered to the naked eye as its typical phenomena. Quite different is the aspect of the inflammatory process if we consider the question, What are the changes in the tissues thus affected? Since the query can be answered only by a study with the microscope, it is plain that the history of inflammation is almost identical with that of histology.

Fifty years ago the symptoms of inflammation apparently coincided with what could be seen with comparatively low powers of the microscope. The web-membrane of the frog was fastened upon a piece of cork, hollowed out. A drop of caustic ammonia was applied, or it was touched with a red-hot glass rod, and the result of the irritation was observed mainly, if not exclusively, in the vascular system of the web-membrane. The current of the blood, which was seen to be normal before the irritation, soon afterwards became irregular and slackened, then oscillating to and fro, and at last arrived at a complete standstill,—the so-called "stasis." Most of the pathologists agreed that by the irritation a rapid contraction of the vessels was produced, soon terminating in a paralysis of the walls of the blood-vessels, which latter did not offer any resistance to the pressure of the blood current. At the same time an inundation of the tissues of the web-membrane with a liquid had occurred, often mixed with extravasated blood, and after twenty-four hours a number of granular corpuscles were seen suspended in the liquid, which was termed "exudate." These corpuscles or cells were thought to have originated in the exudate itself, and were named "exudate-cells." The process of inflammation was evidently a disturbance in the blood and the blood-vessels, and, since all diseases at that time were attributed to a certain mixture or crasis of the blood, the pathologists holding these views were called "humoral" pathologists, with whom all diseases were due to a faulty mixture of the blood,—so-called "dyscrasia."

In order to understand the views of the humoral pathologists, we must take into consideration the discovery of the cells in plants by Schleiden in 1837, and in animals by Theodor Schwann in 1839. In the minds of these observers both the vegetable and the animal organism were composed of cells and their derivations,—viz., intercellular substance. The cells were considered individuals, composed of a vesicle, liquid or semi-liquid contents, and a central nucleus. Millions of such cells were thought to build up an organism. Each cell could originate spontaneously from an albuminous liquid by the formation of, first, the nucleus, and afterwards the cell-wall, in the shape of two watch-glasses held together, and at last an accumulation of liquid within the vesicle, the cell-wall. The pathologists at that time held the opinion that the exudate-cells originated directly in the liquid exudate, which, as a matter of course, came from the blood-vessels, and was considered a modified blood-serum.

The views of the humoral pathologists of the Vienna school, who were guided by Rokitansky, were overthrown, in 1851, by the well-known great man, Rudolph Virchow, at that time in Würzburg, and at present in Berlin. Virchow was the founder of the cellular pathology. He stated[1] that a cell was provided with a wall of its own, held a viscid albuminous liquid, and was the seat of life, which was not so considered by Schwann nor the Vienna school. Virchow held and still holds the views that the organism is composed of a large number of individual cells, separated from one another by an intervening intercellular substance, which he considers to be a product of secretion of the cell. He, however, acknowledged one variety of interconnected cells, termed star-shaped or stellate, such as we know to exist in the myxomatous tissue. With Virchow the vascular disturbances in the process of inflammation were of secondary importance. Instead of an inundation of the tissues with exudate, he admitted only a surplus of nourishing liquid, directly attracted by the cells. He denied the origin of cells from the exudate, but maintained that every cell originated by proliferation from a previous cell. The pus-corpuscles he considered an offspring of the tissue-cells, principally of those of the connective tissue. His assertions were based upon the fact that the tissues lacking blood-vessels, such as the cornea of the eye, could be brought to inflammation and suppuration. The cellular pathology held sway of the minds of pathologists ever since, and is even in our day accepted as the leading doctrine in Europe as well as in this country.

In 1861, Max Schultze,[2] of Bonn, Germany, announced novel views in regard to the construction of cells. He denied the existence of a cell-wall, and said that a cell is a lump of protoplasm

---

[1] "Die Cellularpathologie." Berlin, 1871.

[2] *Müller's Archiv*, 1861.

holding granules and a nucleus, endowed with the properties of life. The word "protoplasm" was accepted by him from the botanist, Hugo von Mohl, who, several years before, applied this term to the contents of vegetable cells. In England, L. S. Beale[1] almost simultaneously announced similar views, applying the term "bioplasma" to the substance which builds up the cells. This bioplasma was the true living or germinal matter, whereas all intercellular substances he considered as inert or formed material. Unfortunately, he was led to term the muscles and nerves "formed material" also, and yet these tissues are known to be the most active of the organism. It is for this reason that Beale's views have attracted but little attention.

Max Schultze's assertions did not directly affect the cell-doctrine, although he admitted that the term "cell" had lost its significance, and may be applied only in honor of its discoverers, Schleiden and Schwann. M. Schultze, at the same time, announced novel views concerning the origin of the intercellular substance, which must be understood in order to trace the development of the modern researches in the process of inflammation. In the views of Virchow the intercellular substance was merely a secretory product of the cells, whereas Max Schultze proved that these substances were derivations of protoplasm. With him a number of protoplasmic bodies become chemically transformed into basis-substance, and thus they are rendered inert, and dead. Beale's doctrine was different, since he said that an originally large mass of bioplasma is converted at the periphery into formed material, and only the central portion remains, forming or germinal matter.

Soon afterwards, E. Brücke, of Vienna, asserted that neither the granules nor the nucleus were essential features of a cell, and consequently he defined the cell as a structureless lump of protoplasm, eventually destitute of granules and a nucleus. The expression "structureless" certainly indicated, not that a structure was absent, but that it could not be made out with the microscope. He likewise admitted that a lump of protoplasm is the real seat of life, and is able to produce new lumps,—*i.e.*, new cells,—by proliferation and division in normal development of the tissues as well as in morbid processes.

Such was the doctrine up to the middle of the seventh decade of our century, when, in 1867, the whole aspect of inflammation became revolutionized by J. F. Cohnheim.[2] This observer saw in the

---

[1] "On the Structure and Growth of the Tissues." London, 1865.

[2] *Sitzungsberichte der Wiener Akademie der Wissenschaften*, 1861.

expanded mesentery of a living frog an emigration of colorless blood-corpuscles through the walls of capillaries and small veins. The observation as such was not novel, since Waller, of England, as early as 1846, had published it without attracting attention. Cohnheim soon afterwards (1869) declared the process of emigration of leucocytes to be the essential feature of inflammation, to such an extent that exudate-corpuscles and pus-corpuscles were to be considered identical with emigrated colorless blood-corpuscles or leucocytes. He denied that the so-called fixed cells of the connective tissue shared in the process of inflammation by proliferation in the sense of Virchow. According to Cohnheim, the tissues, including their cells, simply perish, and the whole inflammatory infiltration is due to an accumulation of leucocytes.

Cohnheim never proved that the emigration of leucocytes is really due to the inflammatory process. On the contrary, it was proved by himself that, in any stagnation of the blood-current, an accumulation of leucocytes occurs inside of the blood-vessels along their walls, followed by an emigration of these corpuscles. It was furthermore proved that the emigrated leucocytes remigrate into the lymph-vessels, and are thus removed from the tissues. Nevertheless, upon the authority of Cohnheim, a large majority of German pathologists accepted the emigration theory. A pupil of Cohnheim, C. Weigert, went so far as to assert that the leucocytes creep into the fixed connective tissue and epithelial cells, thus causing the delusive image of proliferation in them. Many of the younger pathologists have tried hard to show that an aggregation of leucocytes will lead to a new formation of tissue, both in plastic inflammation and in the formation of tumors. All these attempts, however, have proved fallacies, and all reasonable followers of Cohnheim agree in that emigrated leucocytes never produce new tissues.

The most zealous opponent to Cohnheim's views was S. Stricker, of Vienna, who, in a number of accurate observations,[1] has proved, since 1870, that the fixed cells and their coarser offshoots break up into embryonal corpuscles, furnishing the inflammatory infiltration, which is the outcome of proliferation of the tissue-cells themselves. Stricker called all newly-appearing elements "pus-corpuscles," and this nomenclature seems to have greatly enhanced his assertions. In his article on inflammation in "Ashhurst's International Cyclopædia of Surgery," he speaks only of pus-corpuscles as products of inflammation. Still, every practitioner is aware of the fact that

---

[1] *Wiener Medic. Jahrbücher*, 1870–80.

not every inflammation terminates in suppuration; that, on the contrary, many inflammatory processes lead to a new formation of the inflamed tissue,—the so-called hypertrophy or hyperplasia,—and by no means in a destruction of the tissue which is the result of suppuration. Stricker, however, since 1880, has become a convert to the views advanced by his former pupil, Carl Heitzmann. Stricker publicly announced that it required six years of microscopical work before he could confirm Heitzmann's assertions, which he admitted to be adverse to the cellular pathology established by Virchow.

In 1873, Carl Heitzmann, at that time in Vienna, published a series of articles in the Vienna Academy of Sciences[1] with entirely novel discoveries, concerning the structure of protoplasm, the structure and origin of the basis-substance, and the process of inflammation. Since, by personal studies in the laboratory of this observer, I have become convinced of the correctness of his assertions, I take the liberty to explain them more fully than I have done on previous occasions. These views are contradictory to the cell-doctrine and the cellular pathology to such an extent that the latter, to-day, seem to have merely an historical value.

Carl Heitzmann maintains that a cell, or a lump of protoplasm, hitherto considered structureless, is indeed possessed of a pronounced reticular structure. Since he had seen this reticulum in continuous change of place and shape during the locomotions of living protoplasmic lumps, such as amœbæ, colorless blood-corpuscles, etc., he called the substance which builds up the reticulum the living or the "contractile matter" proper. Formations of living matter, according to Heitzmann, are the nucleus, the granules, with their interconnecting threads, and an extremely thin layer, enclosing the lump of protoplasm all around. At first the existence of the reticulum was denied by most observers, and afterwards, when they could not any longer deny its existence, they said that it was not original with Heitzmann, but was discovered by C. Frommann in 1867. Frommann, it is true, speaks of a reticulum in connective tissue and in ganglionic cells, but without giving an illustration thereof. He afterwards declared that he had never used lenses of a higher power than four hundred and fifty diameters. To observe the reticulum in the protoplasm with such a power of the microscope is simply impossible, even to an experienced eye. Eight hundred to one thousand diameters are indeed required for the

---

[1] *Sitzungsberichte der Wiener Akademie der Wissenschaften*, 1873.

study of the reticulum under consideration. That the reticulum exists is to-day a settled fact, the more so as Stricker, in 1890, has succeeded in reproducing it by photography in a living colorless blood-corpuscle of the proteus by means of the electric microscope, with a power of two thousand five hundred diameters.[1] In this photo-micrograph the reticulum in the protoplasm is exactly the same as discovered and described by Carl Heitzmann in 1873.

That the nucleus is made up of living matter became apparently doubtful when, in 1875, the so-called karyo-kinesis of the nucleus was discovered by Strassburger, Bütschli, Flemming, and others. It was shown that the nucleus is composed of loop-like threads representing stars and double-stars preceding its division. Since these loops could be stained deeper by certain analine dyes, especially saffranin, than by the granules of the protoplasm, it was asserted that the nucleus is composed of a substance of its own, called "nuclein and chromatin." It is plain that a substance capable of changing its shape and place must be living matter. The karyo-kenetic threads assumed a deeper color only on account of their being more bulky than the rest of the reticulum in the surrounding protoplasm. Besides, it was shown that even at the height of karyo-kinesis, the loops remain interconnected with the surrounding reticulum of the protoplasm, which again proves their identity. Not only do all movements occur in consequence of contraction and extension of the reticulum, but all new formations and outgrowths start from this substance respectively from the granules, the points of intersection of the reticulum. This again proves that the reticulum is the living matter proper, in the meshes of which there exists a liquid, probably holding nitrogen, but, being a liquid, not endowed with the properties of life.

Originally every so-called cell is a solid granule of living matter, which in turn becomes vacuoled by an accumulation of liquid and, at last, is reticulated, in consequence of perforations of the walls of the vacuoles.

Another discovery of Carl Heitzmann, in 1873, has been that the intercellular, or basis-substance of the connective tissue, is not dead or inert, as hitherto supposed, but is alive in the same sense as the cells themselves. The reticulum of living matter present in the latter is present also in the basis-substance, rendered invisible by chemical changes and a solidification of the originally liquid contents of the meshes in the protoplasm. It was proved that in

---

[1] *Audien aus dem Institute für experimentelle Pathologie*, 1890.

all varieties of connective tissue in the muscles, the nerves, and in the epithelia, the so-called cells are interconnected by means of delicate threads of living matter, or indirectly by the reticulum pervading the basis-substance.

In the development of all varieties of basis-substance the protoplasm shares by process of chemical transformation which renders it more or less firm and solid. It also was proved that in the formation of basis-substance the protoplasm does not perish altogether; but only the lifeless liquid portion which is held in the meshes of the reticulum becomes solidified, whereas the reticulum itself remains unchanged. The correctness of these views are shown in the process of inflammation as well as in the history of the development of the hard tissues of the teeth, such as the dentine, the enamel, and the cementum.

I have dwelt upon the structure and the development of the basis-substance at length, in order to render explicable the process of inflammation as established by Carl Heitzmann, in 1873. He has shown at that time that in inflammation, as well as in the growth of tumors, not only the free protoplasm, the so-called cells of Virchow, participate, but also the basis-substance. It is the living or contractile matter which alone is capable of growth; of an increase in bulk. Since this substance is stored up both in the cells as in the basis-substance, nothing is required but a dissolution or liquefaction of the latter, in order to liberate the living matter which, being reduced to its embryonal condition, shares in the outgrowth of the inflammatory products as much as do the cells themselves.

Inflammation may terminate either in resolution or in hyperplasia, or in suppuration.

*Resolution* occurs when the inflamed tissue is again infiltrated with basis-substance, and thus the previous condition is re-established with such a degree of perfection that no vestige is left of the former morbid process.

*Hyperplasia* appears when, in consequence of the increase of the living matter after the new formation of basis-substance, a certain bulk of tissue is formed in excess of its previous normal amount. In this condition the cells as well as the basis-substance remain interconnected the same as in the production of a normal tissue.

*Suppuration* is the result of the breaking asunder of the interconnections of the protoplasmic bodies from an inflamed tissue which has been reduced to its embryonal condition. As the result of such a separation we observe the appearance of isolated protoplasmic bodies, or pus-corpuscles. Pus, therefore, is a disintegrated tissue, and by no means a dead tissue, as claimed by some. Nothing is dead in the process of suppuration, since every pus-corpuscle remains alive, and, in a fresh condition, is seen to perform amœboid movements, if kept in a fluid not averse to the life of the pus-corpuscle, such as warm urine or serum of blood. Death means necrosis, and every practitioner knows that between suppuration and necrosis there is a pronounced clinical difference, although a necrotic tissue will cause suppuration in its surroundings, but only for the purpose of being eliminated from the living organism.

All these varieties of inflammation occur in both the soft and hard tissues of the teeth and their surroundings. We meet with it in caries, in eburnitis, in the various forms of pulpitis, alveolar abscess, pyorrhœa alveolaris, as well as in exostosis.

At the beginning of 1892, P. Grawitz, professor of morbid anatomy at the University of Greifswald, Germany, published an article,[1] followed by several others, in which he claims to have discovered that in the process of inflammation not only the cells of the connective tissue proliferate, but the basis-substance likewise furnishes a large amount of cells, which he terms slumbering-cells. Grawitz believes that the basis-substance has been developed from cells which remained slumbering until again brought to view by an irritative process, such as inflammation. E. O. Shakespeare, of Philadelphia, in 1882, had previously written of slumbering-cells, which he noticed buried in the basis-substance of the cornea. Quite recently Professor C. Weigert, of Frankfurt, Germany, bitterly attacked Grawitz,[2] calling his discovery "intercellular pathology," in contradistinction to Virchow's cellular pathology. He criticises Grawitz that the latter has ignored the researches of inflammation during the last twenty years, and quotes Stricker as having first discovered that during the process of inflammation the basis-substance likewise produces cells. Weigert, however, still adheres to Cohnheim's emigration-theory, and has never admitted that the connective-tissue cells do proliferate. He tries to explain the images of proliferation by stating that the colorless blood-corpuscles, or leucocytes, creep into the tissue-cells, thus producing the appearance of proliferation. Grawitz admits that he is ignorant of these researches. Weigert, on the contrary, confesses the knowledge of Stricker's publications, but purposely ignored them, because they were adverse to the doctrine of the Cellular Pathology.

---

[1] *Virchow's Archiv. Berliner Wochenschrift.*

[2] *Deutsche Medic. Wochenschrift*, 1892.

It is remarkable that the German pathologists have arrived at discoveries which were made twenty years ago, not by Stricker, as stated, but by Carl Heitzmann, of New York, at that time in Vienna. Stricker himself publicly announced in 1880 that he became convinced of the views of Heitzmann only after six years of hard work. He unquestionably was the first in Europe who acknowledged (twelve years ago) the accuracy of Heitzmann's theory. The discovery, however, is not his.

We, in this country, have been acquainted with Heitzmann's views for the last eighteen years, and I have done my share to prove their correctness and simplicity for the last fifteen years. All this work was simply ignored, especially in Germany, and apparently for no other reason than adoration of Virchow's person and Virchow's views. It is with pride that I can say that we in this country have been ahead of the Europeans for the last eighteen years. It is only recently that they began to realize the truth in the process of inflammation, although they still try to compromise the facts with the doctrine of the cellular pathology, and it will certainly take some time yet before the German pathologists will admit that *the cell-theory* as well as the *cellular pathology* are fallacies.

In conclusion, since most pathologists and dental practitioners seem to be unable to comprehend Heitzmann's views, you will pardon me if I occupy a little more of your time and explain by sketches upon the black-board the formation and dissolution of the basis-substance of connective tissue, bearing directly upon the theories of Virchow, Stricker, and Heitzmann.

## Figures and Tables

**Fig. 1. f1:**
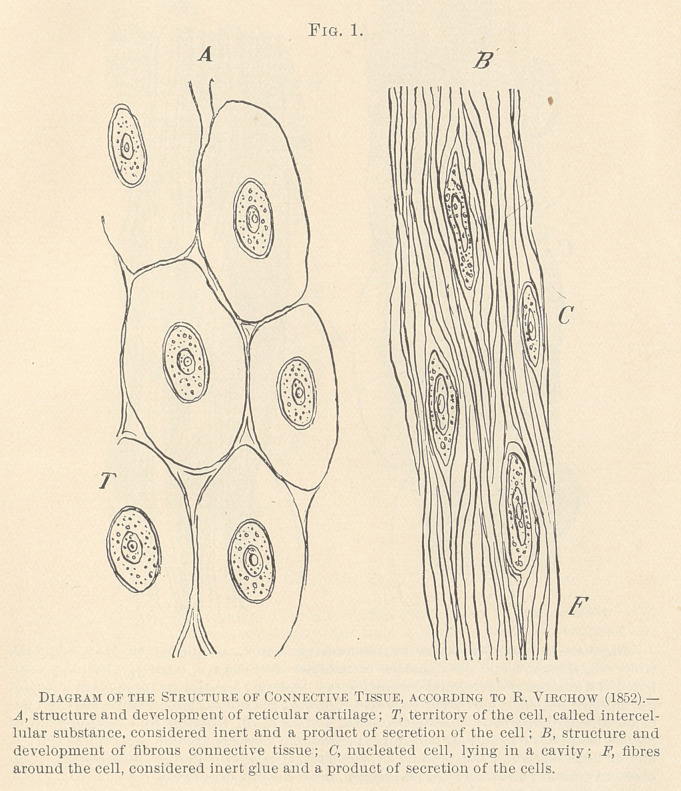


**Fig. 2. f2:**
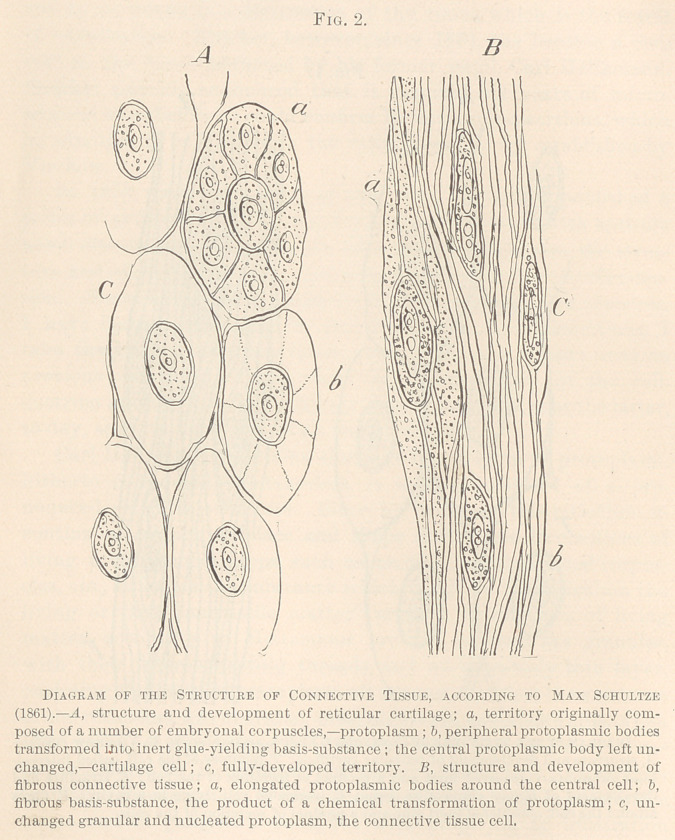


**Fig. 3. f3:**
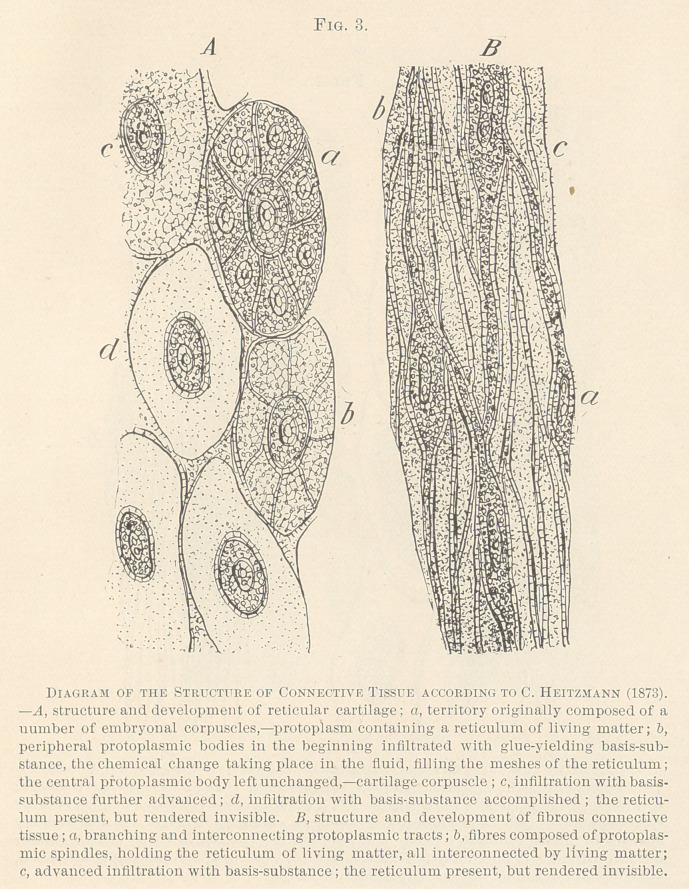


**Fig. 4. f4:**
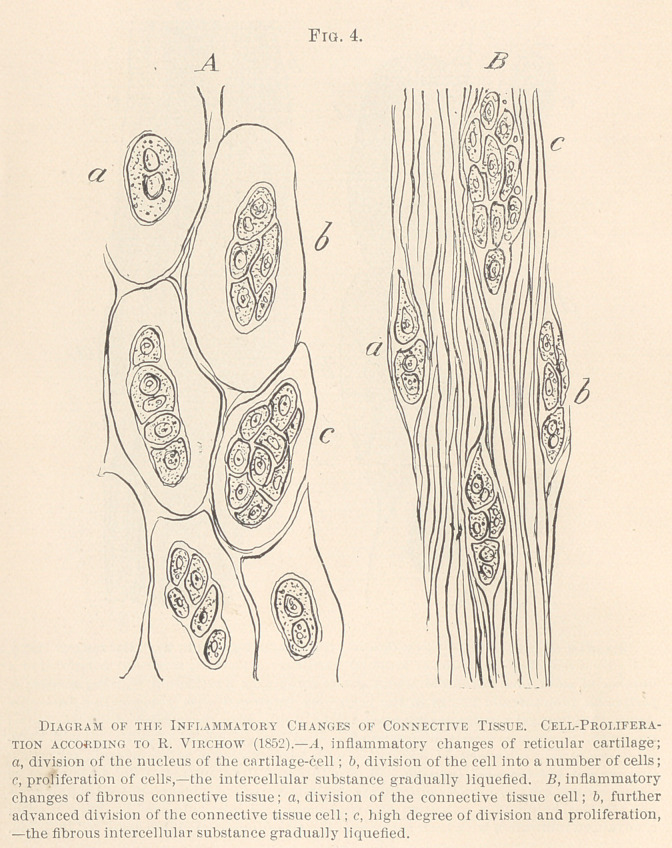


**Fig. 5. f5:**